# The Interplay Between Innate-Like B Cells and Other Cell Types in Autoimmunity

**DOI:** 10.3389/fimmu.2018.01064

**Published:** 2018-05-16

**Authors:** Gregory J. Tsay, Moncef Zouali

**Affiliations:** ^1^Division of Immunology and Rheumatology, Department of Internal Medicine, China Medical University Hospital, Taichung, Taiwan; ^2^College of Medicine, China Medical University, Taichung, Taiwan; ^3^INSERM, U1132, Paris, France; ^4^Université Paris Diderot, Université Sorbonne Paris Cité, Paris, France

**Keywords:** innate immunity, autoimmunity, B-1a cell, marginal zone B cell, innate response activator B cell, T-bet positive B cell, natural killer-like B cell, IL-17-producing B cell

## Abstract

Studies performed in animal models and in humans indicate that the innate arm of the immune system provides an essential role in the initial protection against potential insults and in maintaining tolerance to self-antigens. In the B cell compartment, several subsets engage in both adaptive and innate functions. Whereas B cell subsets are recognized to play important roles in autoimmune diseases, understanding the intricacies of their effector functions remains challenging. In addition to B-1a cells and marginal zone B cells, the B cell compartment comprises other B cells with innate-like functions, including innate response activator B cells, T-bet positive B cells, natural killer-like B cells, IL-17-producing B cells, and human self-reactive V_H_4-34-expressing B cells. Herein, we summarize the functions of recently described B cell populations that can exert innate-like roles in both animal models and humans. We also highlight the importance of the cross talk between innate-like B cells and other adaptive and innate branches of the immune system in various autoimmune and inflammatory diseases. In as much as innate immunity seems to be important in resolving inflammation, it is possible that targeting certain innate-like B cell subsets could represent a novel therapeutic approach for inducing resolution of inflammation of autoimmune and inflammatory responses.

## Introduction

The immune system makes use of two branches of cellular and humoral effectors: the innate and the adaptive arms of immune defense that are able to sense the presence of potential threats and to mount protective immune responses. In the adaptive arm, cells must interact, proliferate, and, over time, generate antigen-specific cells and antibodies, and immune memory. To be effective, the innate arm must be recruited quickly to impart immediate protection, and it is increasingly recognized that cells of the innate branch can enforce protective barrier functions by regulating adaptive immunity. In addition, lymphocytes that differ from conventional lymphocytes in both expression of cell-surface markers, behavior and innate-like characteristics are able to support adaptive immune functions in various ways. This includes innate lymphoid cells (ILCs), natural killer (NK) cells, lymphoid-tissue inducer cells, γδ T cells, natural killer T (NKT) cells, but also B cells.

**Figure 1:**
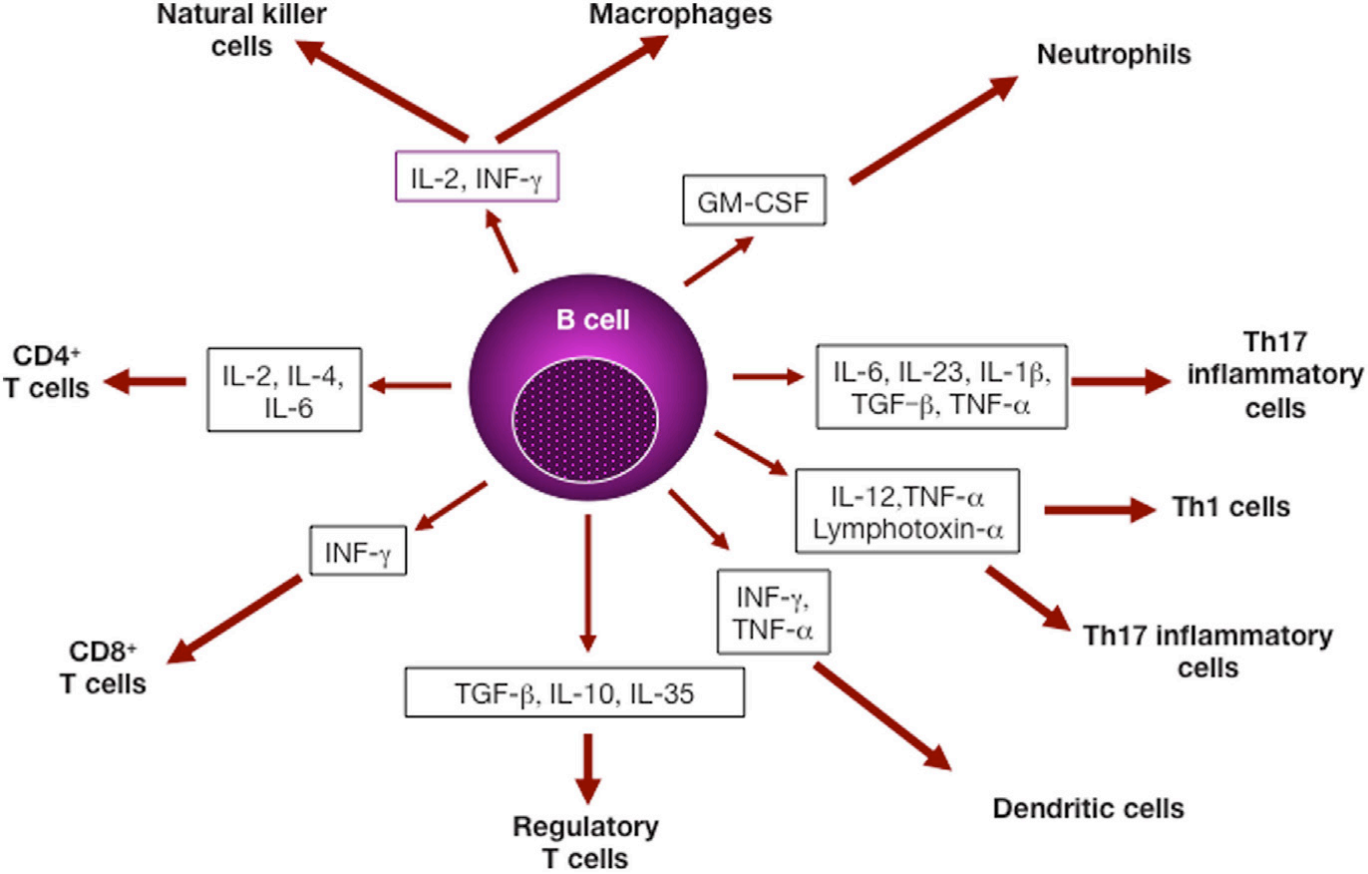
B lymphocytes and cytokine production. Cytokines derived from B cell subsets can impact several cell types of both the adaptive and the immune systems, and affect cell differentiation and/or effector function (3).

In addition to its potential to produce various cytokines (Figure 1), the B cell compartment of the immune system comprises several subsets of innate-like B cells that can produce low-affinity antibody responses able to provide a level of immune protection while follicular (FO) B cells are developed to generate high-affinity antibodies with a lag time of about 5 days (1). As outlined in Table 1, B cell subsets are recognized to play important roles in autoimmune diseases (2). However, understanding the intricacies of their effector functions remains challenging. Herein, we summarize the functions of several B cell subsets that have been described to exert innate-like roles in both animal models and humans. We also highlight the importance of cross talk between innate-like B cells and other adaptive and innate branches of the immune system in various autoimmune and inflammatory diseases.

**Table 1:** Antibody-dependent and independent roles of B cells in autoimmune disorders.

– Production of autoantibodies that that form pathogenic immune complexes – Secretion of autoantibodies that bind *in situ* to target autoantigens – Generation of autoantibodies that act as catalytic antibodies – High autoantigen presentation capacity to T cells – Secretion of pro-inflammatory cytokines and chemokines – Enhancement of dendritic cell antigen presentation ability – Provision of cognate help for autoreactive T cells – Induction of inflammatory Th1 and Th17 cells – Maintenance of T cell memory – Inhibition of regulatory T cells – Organization of tertiary lymphoid tissues and ectopic germinal centers

*B cell subsets are endowed with various functions that can contribute to the generation and/or the amplification of autoimmune diseases*.

## Antibody-Independent Roles of B Cells in Innate Immunity

Neutralizing antibodies produced by B cells are a hallmark of immunity. Less recognized, however, in the fact that B cells also play critical functions as regulators of innate immunity. Initial protection is provided by the innate immune system, including natural antibodies (NAbs), macrophages, NK cells, neutrophils, and cytokines, in particular type I interferons whose production is regulated by lymphotoxin-β derived from hematopoietic cells. Remarkably, splenic B cells represent a copious source of lymphotoxin-β that can act on potentially infected stromal cells to drive IFN-β production, leading to early protection against infection (4). Since these events occur very rapidly after infection, B cells, independently of antibody production, can be considered as key regulators of early innate immunity to various insults. This is, for example, illustrated in studies of vesicular stomatitis virus infection in the mouse. In this experimental model, B cell-derived lymphotoxin-b is required for splenic CD169^+^ macrophage organization and viral capture, further establishing an antibody-independent role for B cells in antiviral immunity (5). Consistently, in the absence of B cells, lymph node macrophages do not allow virus replication (6). In this experimental system, B cells, but not antibodies, are required for macrophage-dependent type I interferon production and for protection against infection. Together with other converging observations, B cells are indispensable for promoting IFN production and protective immunity (7).

## B Cell Subsets in the Peripheral B Cell Compartment

Within the mature peripheral B cell compartment, three distinct subsets that engage in different branches of the immune response have been described: FO B cells, marginal zone (MZ) B cells, and B-1 cells. The majority of mature B lymphocytes are FO recirculating B cells that can home mainly to B cell follicles in secondary lymphoid organs. In follicles located adjacent to T cell zones, FO B cells participate essentially in T cell-dependent (TD) immune responses to protein antigens, but are less responsive to toll-like receptor (TLR) agonists than MZ B cells or B-1 cells.

In contrast to other B cell subsets, which are produced throughout life in the bone marrow, B-1 cells are generated early in ontogeny from progenitors that are present in the fetal bone marrow (8). They represent the major B cell subpopulation in the pleural and peritoneal cavities but are less represented in the spleen (9). B-1 cells are the main producers of NAbs that have been demonstrated to be important in early protection against various infectious insults (10). They respond vigorously to lipopolysaccharide stimulation and take part in the T cell-type-2 independent (TI-2) responses to bacterial capsular polysaccharides (Table 2).

**Table 2:** Functional dichotomy in B cell subsets.

	Adaptive	Innate-like	
	FO cells	B-1a cells	MZ B cells
Recirculation in lymph	+	−	−
T-dependent responses	+	+/−	+
T-independent responses	+	+++	+++
Antigen presentation *in vitro*	+	+++	+++
Favorite isotypes produced	γ1	μ, γ3, α	μ, γ3
Time to peak cell cycle	Long	Short	Short
Proliferation to lipopolysaccharide	+	+++	+++
Proliferation to anti-IgM Ab	+	−	−
CD9 expression	−	+	+

*Shown are the main properties of follicular (FO), B-1a, and marginal zone (MZ) B cells in the mouse*.

This B-1 cell population comprises two subsets: B-1a (CD5^+^) cells and B-1b (CD5^−^) cells that seem to exert distinct functions. For example, in experiments involving rechallenge with *Borrelia hermsii*, B-1b cells, but not B-1a cells, have been shown to generate TI memory B cells (11). After immunization with the model TI-2 antigen NP-Ficoll and the pneumococcal capsular polysaccharide, B-1b cells can also give rise to long-lived memory plasma cells in a thymus-independent manner (12). In addition, they play a prominent role in the immune response against mucosal pathogens and produce IgA in a thymus-independent manner (13, 14).

## Mechanisms That Mediate B-1 Cell Repositioning

Within secondary lymphoid organs, B-1 cells participate in innate-like immune responses. Following a threatening insult, B-1 cells migrate from their initial location, i.e., the peritoneal and pleural cavities, to secondary lymphoid tissues, i.e., the spleen and the lymph nodes, where they secrete antibodies of the IgM isotype. Thus, during influenza virus infection, B-1 cells were demonstrated to relocate to regional mediastinal lymph nodes (MedLN) and to become a primary source of locally secreted IgM (15). To determine the mechanisms that trigger activated B-1 cells to migrate to secondary lymphoid organs and to subsequently differentiate to antibody-secreting cells, the redistribution of these innate-like B cells to regional MedLN was recently investigated in an experimental model of infection (16). The data showed that type I IFN receptor signaling leads to the accumulation of B-1 cells in MedLN, and is facilitated by activation of an integrin family member (CD11b) known to regulate leukocyte adhesion and migration and to mediate inflammatory responses. Specifically, a gradient of innate cytokines elaborated in response to a local threat leads to activation of CD11b on body cavity B-1 cells, promoting their rapid accumulation in inflamed lymph nodes. Since the peripheral blood contains significant numbers of B-1 cells, it is likely that, even in the absence of insults, body cavity B-1 cells represent reservoirs of innate lymphocytes that can rapidly reposition and continuously home to and from body cavities to peripheral tissues *via* the peripheral blood.

## Signals That Drive B1 Cell Homing

The mechanisms that underlie the maturation and expansion of B-1 cells remain under study, but there is evidence that antigen encounters during fetal development lead to positive selection. Studies performed in both wild-type mice and in mice raised in germ-free environments suggest that the selection is triggered by endogenous self-antigens (17). For example, it has been suggested that the repertoire of B-1 cells is selected to bind to evolutionarily important epitopes, such as oxidation-specific epitopes (OSEs) that are a major target of innate NAbs in both mice and humans (18, 19). NAbs represent an important component of innate immunity, and it is generally accepted that they often target OSEs (10, 18).

Oxidation-specific epitopes are neo-self OSEs present on dying cells and damaged proteins that result from the oxidative damage of lipids present in membranes or lipoproteins. Whereas progress has been made in understanding how lipid homeostasis impacts lymphocyte function, the influence of lipid metabolism on B cell-specific responses remains unclear, and the factors that regulate B cell homing into dedicated compartments are not clearly understood. Among the proteins that influence cellular cholesterol homeostasis, the sterol ATP-binding cassette transporter G1 (ABCG1) is an ATPase that promotes unidirectional, net cholesterol efflux to lipoprotein particles. In a relevant study, loss of ABCG1 was found to result in the accumulation of specific oxidized sterols and phospholipids, and to elicit a lung-specific immune response (20). Remarkably, the lungs and pleural cavities of *Abcg1^−/−^* mice contained increased levels of B-1a cells. There was a niche-specific increase in B-1 cells in the lungs and pleural cavities of the knockout mice that was associated with parallel increases in IgM and antibodies that recognize oxidized phospholipid, indicating an increased NAb production. This site-specific expansion of B-1 cells in response to the accumulation of an oxidized lipid antigen could suggest that ABCG1-dependent control of intracellular lipid homeostasis represents a mechanism for the regulation of B-1 cell homing. It is thus tempting to propose that changes in the lipid content of the lung could alter B cell homing pathways. Overall, the demonstration of a niche-specific expansion of B-1 cells in response to oxidized lipid antigens, together with the increase in titers of NAbs that reflect an enhanced innate immunity suggest that loss of ABCG1 results in accumulation of both sterols and phospholipids. Once oxidized, some of these lipids can trigger movement signals for B-1 cells that lead them to home into the lungs and pleural cavity. These oxidized lipids and OSEs could also drive B-1 cell expansion and increased secretion of NAbs.

## Self-Renewal and Repopulation Potentials of B-1a Cells

The origin of B-1a cells remains the focus of investigation with two competing models (8, 21–23). In the “lineage model,” the decision to become either a B-1a or a B-2 cell is made before the expression of surface B cell antigen receptor (BCR). By contrast, in the “selection model,” entry into the B-1a versus B-2 fate starts after BCR engagement, implying that cell fate decision is made after expression of surface IgM and is based on BCR specificity.

To further resolve hematopoietic lineage relationships in B cells, the impact of developmental timing on acquisition of a B-1a potential was recently investigated using cellular barcoding. This innovative biology tool is based on heritable tagging of individual cells with unique DNA identifiers. It allows identification of the progeny of individual cells *in vivo*. The technology is based on heritable tagging of individual cells with unique DNA identifiers. In immunology, it enables simultaneous tracking the burst sizes of multiple distinct responding cells in transplanted animals (24). This experimental approach allowed tracing fetal hematopoietic stem cells (HSCs) clones across serial transplantations. It demonstrated that serially transplantable fetal liver HSCs give rise to B-1a cells as well as to B-2 cells *in vivo*, and that the B-1a potential is lost over time (25). In contrast to other studies suggesting that definitive HSCs lack B-1a potential (26), this observation showing a developmental shift in HSC state links the attenuation of B-1a potential to developmental changes in HSC fate. These developmental changes in lineage potential also indicate that the B-1a potential can be reinitiated by expression of Lin28b, a protein thought to regulate stem cell self-renewal, in a polyclonal fashion that coincides with the reversal to a fetal-like HSC state (25).

In parallel, a transcription factor necessary for the generation and homeostasis of B-1a cells was recently identified (27). This factor, called Bhlhe41, is a member of the basic helix-loop-helix family. It acts as an inhibitor of negative regulators of BCR activation. Whereas Bhlhe41 is important for normal development and function of B-1a cells, it is not essential for that of B-2 cells. In the absence of Bhlhe41, mice show an altered B-1a cell repertoire and a B cell loss that includes lymphocytes expressing self-reactive BCRs known to recognize phosphatidylcholine. In previous studies, the development of B-1a cells had been described to involve multiple positive regulators of BCR signaling, but Bhlhe41 is a remarkable regulator because it is expressed differentially in developing fetal B cells relative to bone marrow B cells. The observation that the survival of B-1a cells with certain BCRs requires the expression of an amplifier of BCR signals, i.e., Bhlhe41, which is highly expressed in fetal lineage B cells as compared with adult lineage B cells, has led to the speculation that cells of the fetal lineage are able to support the maturation of cells with a self-reactive repertoire, but that rearrangement of the same immunoglobulin (Ig) genes in developing B cells derived from HSCs would not survive receptor engagement (23). This proposal accords with the view that the B cell repertoire is critical in determining the phenotype of mature B cells and with the idea that lineage commitment determines cell fate.

## Commitment to the MZ B Cell Fate

Marginal zone B cells occupy a unique positioning in the spleen where they surround the follicles and, hence, are frequently exposed to blood-borne antigens. Such frequent encounters enable MZ B cells to provide immunosurveillance and to shuttle antigens to FO dendritic cells (1, 28). Following exposure to antigens, MZ B cells can present antigen and promote T cell activation, but also differentiate into plasmablasts. Compared with FO B cells, MZ B cells exhibit higher expression of surface IgM, the complement receptors CD35 and CD21, and the lipid antigen-presenting molecule CD1d. Together with an elevated TLR expression, the presence of these receptors allows rapid immune responses to blood-borne pathogens, such as encapsulated bacteria. Gene-expression profiling also allows distinguishing MZ and FO B cells, with differences in their transcriptomes contributing to their differential development, localization and function (29). For example, the transcription factor IRF4 limits the MZ B cell pool size and regulates the positioning of cells in the MZ, and the transmembrane receptor Notch2 seems to be necessary for promoting MZ B cell fate. Because they reside between the marginal sinus and the red pulp of the spleen, MZ B cells are located at the first line of defense against blood-borne particulate pathogens (28). By shuttling between the MZ and the follicles, MZ B cells are able to deliver blood-borne antigen to FO dendritic cells (30). In addition, they can act as potent antigen-presenting cells for the activation of NKT cells by virtue of high expression of the antigen-presenting molecule CD1d (31).

Recent studies revealed that commitment of transitional B cells to the MZ B cell fate is a complex process. Immature B cells receive signals *via* the receptor Notch2 from one of its ligands, Delta-like 1, expressed by fibroblastic reticular stromal cells in the spleen (32). In addition, signals from the BCR are important for accessing the MZ B cell fate (33). Specifically, following BCR signaling *via* the serine-threonine kinase Taok3, the transmembrane metalloprotease ADAM10 translocates from intracellular vesicles to the cell surface. This translocation leads to a cleavage step that frees the intracellular domain of Notch, and the fragment generated can translocate to the nucleus where it will drive the MZ cell fate-determining transcriptional program (33). In addition to Taok3 signaling (33), the RNA-binding protein ZFP36L1 was identified to play an indispensable role in determining the identity of MZ B cells by promoting their proper localization and survival (34). In its absence, MZ B cells are mislocalized and die. Further investigation is required to determine whether such mechanism could contribute to shaping of the B cell repertoire.

## MZ B Cell Fate and Immune Tolerance

This molecular pathway that links BCR signaling and Notch2 signaling in driving the MZ B cell fate after contact with stromal cells expressing a Notch ligand (33) could have advantages in shaping the MZ B cell repertoire. For example, autoreactive immature B cells that encounter self-antigen, but do not rapidly come into contact with the appropriate stromal cells, would not develop into MZ B cells. By contrast, B cells reactive with non-self-antigens, i.e., microbiota-derived antigens that can be present in the peripheral blood, would be exposed to both proper BCR and Notch2 signaling, which would drive them into the MZ B cell fate. Such a mechanism would be advantageous for the immune system because it would provide protection against microbial infection and allow negative peripheral selection of autoreactive B cells.

Since the gut is a key interface between the immune system and the environment, it has become clear that the mucosal immune system of the gut plays a chief role in maturation of the immune system, in shaping the T cell and B cell repertoires, and in induction of immune tolerance to food antigens and to microbiota-derived antigens. This recent insight is being used in investigation of the pathogenesis of various diseases, including type-1 diabetes (T1D). Gut microbes are known to release the short-chain fatty acids (SCFAs) acetate and butyrate from specialized diets. Since the NOD mouse develops symptoms of spontaneous diabetes with a number of similarities to human T1D, it can be used to investigate the importance of the SCFA microbial metabolites acetate and butyrate in this autoimmune form of diabetes. A recent comprehensive study disclosed that SCFA-rich diets had substantial effects on the immune system through different modes of action (35). Interestingly, the acetate diet resulted in a decreased number of splenic B cells, particularly transitional and MZ B cells. By contrast, the butyrate diet was associated with an increased number and function of regulatory T cells. While the SCFA-enriched diets had a clear effect on B cells, especially MZ B cells, which were markedly reduced in number and function (35), it remains unknown whether SCFAs had an impact on plasma cells or on mucosal IgA-producing B cells. Further investigation of these beneficial effects could have potential therapeutic applications, not only in this form of diabetes, but also in other autoimmune diseases.

## Multi-Way Interactions among Human Innate Cells

The phenotype of MZ B cells differs in mice versus humans. In the mouse, MZ B cells form a population of IgM^+^IgD^lo^CD^21hi^CD23^lo^ B cells that do not recirculate and that are thought to be a lineage separate from FO B cells. By contrast, human MZ B cells recirculate and are often somatically hypermutated, suggesting a memory B cell origin. However, high-throughput V_H_ sequencing of human B cell subsets indicates that IgM memory and MZ B cells constitute two distinct entities (36). Given such differences, it remains unclear if MZ B cells play similar conserved roles in both species.

For their activation, MZ B cells rely on unconventional sources of stimulation, such as TLRs, which contrasts with the requirement of FO B cells. To become fully activated, these latter cells receive a first signal delivered trough antigen recognition by the BCR followed by a T cell-derived second signal. In fact, it appears that there is a complex set of intercellular interactions that facilitate antibody production by MZ B cells in both TI type 1 settings and TI type 2 settings, and that require a small subset of ILCs that reside mainly in the MZ of the human spleen (37). These cells express CD127 (IL-7 receptor), CD117 (stem-cell factor receptor), and the transcription factor RORγt. In response to IL-1β and IL-23, they produce IL-22, which places them in the mucosal cell-like ILC3 category. These MZ ILCs are located in the vicinity of stromal cells expressing the integrin ligand MAdCAM-1, suggesting that they represent the human equivalent of mouse marginal reticular cells (MRCs) that express TLR3, TLR4, and TLR9.

Intriguingly, interactions between MRCs and ILCs can synergistically amplify a signal from TLR ligands that enhances human MZ B cell activation and differentiation into antibody-secreting cells (37). Furthermore, neutrophils that reside in the MZ can be activated by ILCs, which will further boost MZ B cell stimulation (38). These observations have potential implications for our understanding of immune tolerance to self. Since these events take place in the absence of BCR engagement, they are reminiscent of polyclonal TI type 1 responses. This cellular interactive web could lower the threshold of MZ B cell activation to TI antigens, which would increase their responsiveness to foreign threats and simultaneously maintain tolerance to self-antigens.

## T-bet^+^ Age-Associated B Cells (ABCs)

A novel subset of B cells, termed ABCs, has recently been identified in mouse models. ABCs express high levels of CD11c and the transcription factor T-bet, which distinguishes them from other B cell subsets (39). Subsequently, T-bet was found to be necessary and sufficient for the appearance of this subset, and triggering of the BCR, IFN-γ receptor, and TLR7 on B cells induces high levels of T-bet expression. Since ABCs exhibit a unique T-bet driven transcriptional program, they differ substantially from other B cell subsets in their activation requisites, functional capacities, and survival requirements (40). They respond poorly to BCR engagement, but survive, which distinguishes them from FO and MZ B cells. Remarkably, ABCs express the canonical BAFF receptors BR3 and TACI, but do not rely on BAFF for survival, another feature that distinguishes them from FO and MZ B cells. Following simulation with either TLR9 or TLR7 agonists, either alone or in combination with BCR ligation, they proliferate vigorously and produce the regulatory cytokines IL-10 and IFN-g. Overall, these functional properties suggest that ABCs could have profound effects on the dynamics and homeostasis of peripheral B cell subsets.

Since ABCs are potent antigen-presenting cells, they could play a role in autoimmune responses by presenting self-antigen to autoreactive T cells. Consistently, T-bet^+^ ABCs appear in autoimmune-prone mice and in autoimmune patients. Thus, several groups have reported the presence of T-bet-expressing B cells in autoimmune-prone mice and in autoimmune patients, including rheumatoid arthritis (RA) and scleroderma patients (41). B cells with a similar phenotype (CD21^−^CD19^hi^CD11c^+^) have also been found to be enriched in the blood of common variable immunodeficiency patients with autoimmune cytopenia, systemic lupus erythematous (SLE), RA, and Sjögren’s syndrome. Importantly, several groups have recently reported the appearance of T-bet^+^ B cells in autoimmune patients suffering from SLE (42), multiple sclerosis (43), and Crohn’s disease (44). More recently, investigators demonstrated that T-bet expression in B cells is critical for the rapid initiation and progression of autoimmune responses, end-organ damage, and early mortality during lupus-like autoimmunity (45). They also have shown that ABCs are located at the T cell/B cell border in the spleen, which could increase the likelihood of contact between Ag-specific T cells and antigen-presenting ABCs. Remarkably, their BCRs are autoreactive, suggesting that ABCs are precursors for autoantibody production. All these features indicate that ABCs will be ideal presenters of autoantigens to T cells. Together, these observations suggest that T-bet-dependent activation of autoreactive B cells is important for the development and/or the progression of human autoimmunity, and it has been suggested that T-bet^+^ B cells represent promising targets for treatment of human autoimmunity (39). However, other investigators found that during the remission phase of collagen-induced arthritis (CIA), an experimental model for RA, MZ B cells express an elevated level of T-bet (46), thereby confirming the existence of IL-10/T-bet co-expressing cells. These observations, suggesting that T-bet could contribute to the remission of CIA by facilitating the regulatory potential of IL-10^+^ MZ B cells, does not militate in favor of targeting T-bet^+^ B cells for therapeutic purposes. They also raise questions regarding the functional relationships between T-bet^+^ B cells, MZ B cells, and regulatory B cells.

## NK-Like B Cells

Recently, a separate subpopulation of innate B cells, termed NKB cells, was identified in mice, and in human (47). They exhibit CD19^+^NK1.1^+^ signature markers and reside mainly in the spleen and mesenteric lymph nodes. Their identity seems unique and is distinct from that of NK and B cells. NKB cells can produce large amounts of interleukin-18 (IL-18) and IL-12, and, consequently, are able to activate type 1 innate lymphoid cells (ILC1s) and NK cells to initiate innate immunity against invading microorganisms. Unlike other cell lineages, NKB cells are postulated to harbor unique fate-decision transcription factors that could specifically drive their progenitors to differentiate into mature NKB cells (47). It is possible that NKB cells act as a separate subset of innate B cells and play a critical role in the early stage of innate immune responses.

Reminiscent of B-1 cells and MZ B cells, which express a limited diversity of germline-encoded BCRs and are rapidly activated upon challenge with innate stimuli, NKB cells exhibit a non-Gaussian distribution of the length of their Ig heavy-chain third hypervariable region (47), suggesting that NKB cells display a restricted BCR repertoire. The fact that they harbor a low-diversity BCR repertoire different from that of B cells suggests a restricted recognition of antigens. However, the antigen repertoire of NKB cells needs to be better delineated to determine if they can recognize self- and non-self-antigens. In addition, further studies are required to determine whether these NKB cells represent a *bona fide* separate subset of innate B cells that are distinct from conventional B cells and to decipher their potential role in inflammatory and autoimmune conditions. Since NKB cells can produce substantial amounts of IL-18 and IL-12 that lead to activation of innate lymphocytes, and in as much as IL-18 is also an inflammatory factor responsible for promotion of autoimmune diseases, future studies should investigate whether NKB cells are implicated in the pathogenesis of autoimmune diseases.

## Human Innate-Like, Self-Reactive V_H_4-34-Expressing B Cells

In humans, V_H_4-34-B cell clones expressing the germline Ig variable heavy-chain 4-34 (V_H_4-34) gene are common in the naive B cell repertoire, but are rarely found in IgG memory B cells from healthy individuals. Several groups showed that the V_H_4-34 gene codes for autoantibodies that recognize I/i carbohydrates expressed by red blood cells with a specific motif in their framework region 1 (FWR1) (48, 49). In recent studies of patients exhibiting a genetic deficiency in IRAK4 or MYD88, which mediate the function of TLRs except TLR3, CD27^+^IgG^+^ B cells comprised V_H_4-34-expressing clones and showed decreased somatic hypermutation frequencies (50). Importantly, whereas V_H_4-34-encoded IgGs from healthy donors harbored FWR1 mutations abrogating self-reactivity, their counterparts from IRAK4- and MYD88-deficient patients often displayed an unmutated FWR1 motif, which enables these antibodies to recognize I/i antigens present on erythrocytes. Paradoxically, this self-reactivity was associated with a potential of these V_H_4-34-encoded IgG clones to bind commensal bacterial antigens.

It is possible that germline-encoded self-reactive V_H_4-34^+^ antibodies recognizing I/i carbohydrates expressed on erythrocytes and the corresponding B cell clones exert beneficial functions through their cross-reactivity with antigens present on commensal bacteria that reach the circulation. This view is in line with the proposal that B cells expressing germline-encoded self-reactive V_H_4-34 antibodies may represent an innate-like B cell population specialized in the containment of commensal bacteria when gut barriers are breached (50). Other studies described human V_H_4-34-expressing B cells that are anergic, a state that precludes them from being recruited into B cell follicles (51–53). Consistently, the fact that the majority of V_H_4-34-expressing IgG^+^ B cells isolated from healthy donors were described to acquire mutations that abolish self-reactivity to I/i antigens (50) supports the contention that V_H_4-34^+^ B cells are *bona fide* anergic. It will be important to determine the interrelationship between the innate-like function and the anergic state of human V_H_4-34^+^ B cells.

## IL-17-Producing B Cells and Innate Immunity

Other investigations have identified B cells as a chief source of rapid, innate-like production of IL-17 in response to infection, and, remarkably, IL-17^+^ B cells outnumbered inflammatory Th17 cells (54). In addition, the IL-17^+^ B cells had a plasmablast phenotype (CD19^+^B220^dim^GL7^−^CD138^+^), suggesting that, in addition to antibody secretion, IL-17 production may represent an additional key effector function for plasma cells during infection. Consistent with that idea, B cell-intrinsic production of IL-17A was required for efficient control of parasitemia and regulation of inflammatory responses. The IL-17 B cell production in response to infection occurred *via* a previously unknown pathway involving a trans-sialidase. Through the use of inhibitors and genetic models, a requirement for both Src and Btk-Tec kinases was demonstrated in B cells (54). Genetic deletion or pharmacological blockade of CD45 abrogated IL-17 B cell production, suggesting that the trans-sialidase can trigger CD45 compartmentalization or oligomerization, and initiate an IL-17 transcriptional program that operates independently of key candidate receptors on the B cell surface. It is possible that this enzyme is able to modify the B cell-surface protein CD45, triggering Src and Btk kinase intracellular signaling. Importantly, this signaling program operated in both mouse primary B cells and human primary B cells.

Overall, these observations may indicate that the generation of IL-17^+^ B cells represents an unappreciated arm of the innate immune response required for pathogen control. Since both B cells and IL-17 have been linked to a range of autoimmune diseases, it will be important to determine the potential role(s) of IL-17^+^ B cells in candidate autoimmune disorders.

## Innate Response Activator (IRA) B Cells

In a related experimental study, peritoneal B-1a cells were found to give rise to a population of B cells, called IRA B cells, that produce the growth factor GM-CSF (55). This IRA B cell population arises in the mouse peritoneum and accumulates in large numbers in the splenic red pulp. In additional studies, pleural B cells were demonstrated to migrate from the pleural space and to relocate to the lung parenchyma where they produce abundant natural IgM Abs that bind to bacteria and neutralize them (56). It is of note that the Abs that recognize oxidized phospholipids (20) also bind *S. pneumoniae* and provide optimal protection to mice from this pathogen (56). This innate immune mechanism is able to clear bacteria and to protect against pneumonia, and the B cell-derived GM-CSF is the autocrine instructor required for IgM production. This early appearance of a unique GM-CSF-producing B cell is intriguing. It suggests that IRA B cells are able to educate other cell subsets, such as myeloid cells. It will be important to elucidate the precise molecular and cellular events that link GM-CSF signaling to IgM production.

These observations may have implications toward our understanding of the pathogenesis of autoimmune diseases. A growing body of evidence suggests that autoimmunity in patients is initiated outside the tissue that is targeted by the autoimmune attack. In RA, for example, serum autoantibodies are detectable years before the development of the initial joint symptoms, and mucosal tissues, including the lung and the oral cavity, have been implicated as potential initiating sites for disease development (57).

As occurs in other organs, immunologic lung diseases develop when the normal mechanisms of immune self-tolerance are disrupted. In the lung, macrophages and lymphocytes are the key cells involved in the initiation and perpetuation of undesirable immune responses. Macrophages can ingest and degrade the inhaled antigens and serve as scavenger cells. In addition, they can act as antigen-presenting cells for T cells. Even tough lymphocytes are present in low numbers in the normal lung parenchyma, a subset of lymphocytes that have been triggered by relevant antigens in the surrounding lymphoid tissues are able to migrate to the lung and participate in inflammatory responses. This could account for the fact that systemic autoimmune diseases frequently involve the lung, the pleura, pulmonary parenchyma, or airway. The observation that pleural space B cells control the early responses to insults broadens our understanding of the immune system’s spatio-cellular dynamics. The identification of the GM-CSF–IgM axis could have implications for our understanding of autoimmune diseases in human.

## The Interplay Between Innate-Like B Cells and Other Cell Types in Autoimmunity

B lymphocytes are known to exert crucial non-redundant roles in the innate and adaptive arms of the immune system through both antibody-dependent and antibody-independent mechanisms. For example, during acute infection, B lymphocytes play a role in the early innate immune response, where they aid in mounting an efficient and protective inflammatory response (55). Even in the inflammatory response secondary to other forms of acute injury, experimental studies uncovered a key role for B cells in production of CCL7, previously called monocyte-chemotactic protein 3, or MCP3 (58). In that setting, B cells are able to produce Ccl7 and to induce a Ly6C^hi^ monocyte mobilization from the bone marrow and recruitment to the heart, leading to enhanced tissue injury and deterioration of myocardial function. Remarkably, high circulating concentrations of CCL7 and the B cell longevity factor BAFF in patients with acute myocardial infarction predict increased risk of death or recurrent myocardial infarction (58). The precise B cell subset involved in this setting remains to be examined in more detail. However, the results indicate that this pathogenic effect of B cells depends in part on BAFF-R signaling, a finding reminiscent of observations showing that BAFF-R deficiency or B cell depletion can reduce the development of atherosclerotic lesions in several experimental models (59). Since atherosclerosis can be considered an auto-inflammatory disease associated with inflammatory factors characterized by lipoprotein metabolism alterations that lead to immune system activation with the consequent proliferation of smooth muscle cells, narrowing arteries, and atheroma formation, the contribution of various B cell subsets to post-ischemic injury is likely to be important.

During atherosclerosis, several studies consistently demonstrated that B lymphocytes play prominent roles (60, 61). Thus, natural IgM antibodies derived from B-1 cells have been found to be atheroprotective, and B-2 cell responses were demonstrated to promote atherogenesis chiefly by supporting proatherogenic T cells. The contribution of adaptive FO B cells to the development of atherosclerosis can be inferred from their roles in the support of proatherogenic T follicular helper (TFH) cells and germinal center responses. In addition, the contribution of innate-like MZ B cells to regulation of the immune response in atherosclerosis was investigated in mice. In that setting, MZ B cells were found to activate a homeostatic program in response to high-cholesterol diet (HCD) and to regulate the differentiation and accumulation of TFH cells (62). In mice fed with a HCD diet, MZ B cells upregulated surface expression of the immunoregulatory ligand PDL1 and increased the interaction between MZ B cells and pre-TFH cells, which led to PDL1-mediated suppression of TFH cell motility, alteration of TFH cell differentiation, reduced TFH abundance and suppression of the proatherogenic TFH response. This MZ B cell role in controlling the TFH-germinal center response to a cholesterol-rich diet is critical in limiting exaggerated adaptive immune responses and in substantially reducing the development and progression of atherosclerosis (62). In the absence of MZ B cells, there is an excessive accumulation of suboptimally differentiated TFH cells and increased levels of T helper and T effector memory cells. However, the signals that instruct MZ B cells to leave the MZ and guide them to TFH cells remain unclear.

Similarly, various cell types play a role in the autoimmune disease T1D, but the trigger mechanisms involved in the early stages of disease pathogenesis remain under investigation. In early studies, the number of circulating CD5^+^ B cells was reportedly higher in children with recent onset T1D, as compared with patients with long-term disease or controls (63). In addition, as has been observed in other autoimmune disease, an altered BCR signaling threshold has been disclosed in patients with T1D as compared with healthy controls (64, 65). In diabetes-prone NOD mice, an elevated frequency of self-reactive B cells is detectable, as compared with C57BL/6 and BALB/c mice (66), and peritoneal B-1a cells were demonstrated to participate in T1D development in NOD mice (67). In further studies of young female NOD mice, physiological beta-cell death was found to induce the recruitment and activation of B-1a cells, neutrophils, and plasmacytoid dendritic cells (pDCs) to the pancreas (68). Experiments based on depletion of cell subsets indicated that B-1a cells, neutrophils, and IFN-α-producing pDCs are required for the initiation of the diabetogenic T cell response and T1D development (68), suggesting that an innate immune cell dialog that starts in the pancreas of young NOD mice can lead to the initiation of T1D. This IFN-α production would create an inflammatory milieu favorable for a diabetogenic adaptive response that leads to autoimmune diabetes. It is possible that the interplay between B-1a cells, neutrophils and pDCs represents a common feature of other autoimmune diseases.

## Discussion

Studies performed in animal models and in humans indicate that the innate arm of the immune system provides an essential role in the initial protection against potential insults and in maintaining tolerance to self-antigens. Investigations of innate-like lymphocytes, including γδ T cells and NKT cells, suggest that there are no tight boundaries between innate and adaptive immunity. As discussed above, the existence of several B cell subsets with distinct effector functions enables production of pathogenic autoantibodies and promotion of inflammatory cascades that involve various other cell types (Table 3). Within inflammatory lesions, there is a complex and dynamic cross talk between B cells and other cell types.

**Table 3:** Principal B cell subsets with innate-like functions.

B cell subset	B-1a cells	Marginal zone B cells	T-bet positive B cells	Innate response activator B cells	Natural killer-like B cells	IL-17-producing B cells	Human self-reactive V_H_4-34-expressing B cells
Associated pathology	Type-1 diabetes (T1D), rheumatoid arthritis (RA)	Atherosclerosis, T1D	RA, scleroderma, systemic lupus erythematous	Under investigation	Under investigation	Under investigation	Under investigation

In as much as innate immunity seems to be important in resolving inflammation (69), it is possible that targeting certain innate-like B cell subsets could represent a novel therapeutic approach for inducing resolution of inflammation of auto-inflammatory responses. However, it remains uncertain whether observations made in mice will translate meaningfully to the extent of human subjects. In addition, some of the newly described B cell subsets need a more detailed characterization using novel methodical approaches.

Throughout the years, categorization of B cells has relied on immunophenotyping by flow cytometry, which allows identification of cells in suspension by expression of cell-surface receptors and intracellular cytokines. However, the census of B cells remains incomplete because, depending on environmental encounters, each B cell can be at a different stage of activation or differentiation. In addition, the same cell subset can be present in different locations of the organism, but in distinct phenotypes that result from adaptation to the tissue of residence. Furthermore, B cells are endowed with particular antigen receptor sequences, and, therefore, are clonal in nature, which creates a unique genetic diversity into these cells.

In recent years, development of single-cell genomics and spatial profiling methods is enabling genome-wide quantification of molecules in thousands of individual cells, and multiplex spatial analysis of proteins and RNA *in situ* (70). These new approaches can quantify subtle changes in gene expression between individual cells and dynamic expression modifications during an immune response. The power of single-cell genomics to identify previously unidentified subpopulations is illustrated by recent studies of cells of the immune system. For example, studies using human DCs have revealed previously unknown DC subsets and provided insight into the complexity of the lineage of these cells (71, 72). In parallel, similar approaches led to characterize variations within Th17 cells, and to describe a spectrum of cell subsets with distinct levels of pathogenicity, that, upon adoptive transfer, are able to induce symptoms of autoimmune disease in animal models (73, 74).

For B lymphocytes, single-cell techniques are ideal to study the antigen receptor repertoire, and its relation to the B cell subtype and state. It is likely that these new approaches will lead to a more precise characterization of B cell subsets and their effector functions. They should considerably enhance our understanding of the complexity of the B cell compartment and its innate-like functions within the immune system, and beyond. It is possible that combining B cell repertoire analysis, single-cell genomics, new emerging spatial approaches, and multiplex immunophenotyping could lead to a novel generation of diagnostics and therapeutic approaches.

## Ethics Statement

The work is exempt from ethical approval procedures.

## Author Contributions

Both authors made substantive intellectual contributions to this study to qualify as authors. MZ designed and drafted the manuscript. GT contributed discussion and re-drafted parts of the manuscript.

## Conflict of Interest Statement

The authors declare that the research was conducted in the absence of any commercial or financial relationships that could be construed as a potential conflict of interest. The handling Editor declared a shared affiliation, though no other collaboration, with one of the authors MZ.
